# Survey of canine use and safety of isoxazoline parasiticides

**DOI:** 10.1002/vms3.285

**Published:** 2020-06-02

**Authors:** Valerie Palmieri, W. Jean Dodds, Judy Morgan, Elizabeth Carney, Herbert A. Fritsche, Jaclyn Jeffrey, Rowan Bullock, Jon P. Kimball

**Affiliations:** ^1^ Momentum Consulting, LLC Monroe CT USA; ^2^ Hemopet Garden Grove CA USA; ^3^ Dr. Judy Morgan's Naturally Healthy Pets Woodstown NJ USA; ^4^ Peaceful Pet Passages York PA USA; ^5^ Fritsche Consulting Services Navasota TX USA; ^6^ Allegiant Weston CT USA; ^7^ Independent Contractor Austin TX USA; ^8^ The Potter‐Hawkins Group LLC Chapel Hill NC USA

**Keywords:** fleas, isoxazoline, neurotoxicity, parasiticide, ticks

## Abstract

A veterinarian and pet owner survey (Project Jake) examined the use and safety of isoxazoline parasiticides given to dogs. Data were received during August 1–31, 2018 from a total of 2,751 survey responses. Forty‐two percent (1,157) reported no flea treatment or adverse events (AE), while 58% (1594) had been treated with some parasiticide for flea control, and of those that received a parasiticide, the majority, or 83% (1,325), received an isooxazoline. When any flea treatment was given, AE were reported for 66.6% of respondents, with no apparent AE noted for 36.1%. Project Jake findings were compared to a retrospective analysis of publicly available Food and Drug Administration (FDA) and European Medicines Agency (EMA) reported AE. The number of total AE reported to FDA and EMA were comparable, although a 7 to 10 times higher occurrence of death and seizures was reported from the EMA or from outside the United States (US). Serious AE responses for death, seizures and neurological effects reported in our survey were higher than the FDA but moderately lower than the EMA reports. These sizable global data sets combined with this pre‐ and post‐parasiticide administration survey indicated that isoxazoline neurotoxicity was not flea‐ and tick‐specific. Post‐marketing serious AE were much higher than in Investigational New Drug (IND) submissions. Although the labels have recently been updated, dogs, cats and their caregivers remain impacted by their use. These aggregate data reports support the need for continued cross‐species studies and critical review of product labelling by regulatory agencies and manufacturers.

## Graphical abstract

A veterinarian and pet owner survey (Project Jake) examined the use and safety of isoxazoline parasiticides given to dogs during August 1‐31, 2018 from 2751 survey responses. When any preventive treatment was given, AE were reported for 66.6% of respondents, with no apparent AE noted for 36.1%. These survey data support the need for continued cross‐species studies and critical review of product labeling by regulatory agencies and manufacturers.

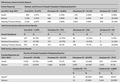

## 1 INTRODUCTION

The isoxazoline class of compounds are ligand‐gated ion channel inhibitor parasiticides that have been developed for at least 20 years (Garcia‐Reynaga, Zhao, Sarpong, & Casida, 2013; Nakata et al., 2017; Ozoe, Ozoe, Nakahira, & Mita, 2010; Quan et al., 1999; Shoop et al., 2014; Zhao & Casida, 2014). When initially designed and synthesized as a series of bisbenzamidine isoxazolines by Dupont Pharmaceuticals, their purpose was to act as antithrombotic agents that inhibit the activity of coagulation factor Xa, although side effects included excessive bleeding and hemolysis (Quan et al., 1999). The earlier reports of these synthesized compounds and their mechanisms of action laid the groundwork for subsequent pre‐clinical studies as parasiticides, which were described in a special issue of Veterinary Parasitology in 2014 (Drag, Saik, Harriman, & Larsen, 2014; Shoop et al., 2014). Subsequent to their development as parasiticides they are now widely used throughout the world and are considered by most to be efficacious and safe, even in 8‐week old puppies (Drag et al., 2014; Kuntz & Kammanadiminti, 2017). However, despite the estimated millions of doses being given, adverse events (AE) that had previously been believed to be rare (Gaens, Rummel, Schmidt, Hamann, & Geyer, 2019; Quan et al., 1999), post‐marketing AE have been increasingly reported since the release of these products.

More recently, Gaens et al. (2019) reported suspected transient neurotoxicity in a 7‐month old female Danish spaniel (Kooikerhondje) puppy. About 24 hr after administering fluralaner, she exhibited generalized ataxia, myoclonic jerks, tremor of the head and body, muscle twitching and oral dysphagia; she fully recovered after 10 hr without treatment (Gaens et al., 2019). Factors including genetic predisposition that could predispose an individual animal to AE are unknown, although the effects of an MDR1 efflux carrier at the blood‐brain barrier could play a role (Saidijam, Dermani, Sohrabi, & Patching, 2017).

The impetus for the present survey inquiry into the potential AE associated with the use of canine flea and tick treatments was prompted by non‐AE documented reports and clinical experiential AE reports from veterinarians and pet owners worldwide. These reports were apparently disregarded or failed to generate concern and responsiveness from the manufacturer(s), which was compounded by the failure to report serious AE on their package labelling, until recent label changes were made from 2018 to 2019. Consequently, the present team of experts was formed to gain an objective understanding of any toxic effects that may be related to the isoxazolines**.**

Isoxazolines exhibit killing activity through effects on the nervous system. Isoxazoline efficacy is based on their modality as non‐competitive GABA (gamma‐aminobutyric acid) receptor antagonists that are putatively much more selective for GABA receptors in fleas or ticks, than for those in mammals, including humans (Gassel, Wolf, Noack, Williams & Ilg, 2014; Shoop et al., 2014). They bind to arthropod gamma‐aminobutyric acid (GABA)‐gated chloride channels (GABACls) in nerve and muscle cells, which blocks the transmission of neuronal signals. Some of the currently registered isoxazolines claim an adequate safety margin for canine and feline administrations and that the mechanism of action is insect‐specific. However, although some recently marketed isoxazoline package inserts do mention seizures or other neurotoxic effects, these effects, including death, are not insect‐specific. The purpose of the current study was to perform an independent survey to document the use and safety of the isoxazoline parasiticides pre‐ and post‐administration.

## 2 METHODOLOGY

### 2.1 Survey

A Survey questionnaire instrument was developed in conjunction with the Project Jake team. It was distributed electronically by mail throughout the United States to veterinarians, veterinary clients, pet caregivers/owners, kennel club groups and on social media sites between August 1 and 31, 2018. The questionnaire addressed the respondent's use of a parasiticide for flea and/or tick control on their dogs pre‐ and post‐administration. We chose to focus just on the dog initially rather than including cats. While a reporting bias can arise for participants of this type of survey, respondents were encouraged to complete initial questions even if they had not seen or were unaware of any AE from the use of isoxazoline or other flea or tick preventive compounds. Pitfalls of electronic online surveys include: absence of interviewer input, may not effectively reach the intended population, and survey bias or fraud (Evans & Mathur, 2018; Nayak & Narayan, 2019; Rice, Winter, Doherty, & Milner, 2017; Vaske, 2011). The authors acknowledge these potential shortcomings of the survey instrument, but believe that the findings described herein reflect valid concerns. In addition to the survey data shown here, the findings were compared to the reported FDA and EMA data. Freedom of Information Act (FOIA) requests were sent to the FDA concerning their isoxazoline AE reports (see Appendix, # 1–8). For the EMA reports, the findings reported by the Committee for Medicinal Products for Veterinary Use (CVMP) at the meeting of 5–7 September 2017 were used. These represented the CVMP Cumulative Reports from Jan 2013 to Sept 2017 (see Appendix, # 9). An analysis of the occurrence of AE was conducted to compare the number of Project Jake AE, with the FDA and EMA AE pharmacovigilance reports, as listed in the Appendix, #1‐9.

### 2.2 Data analysis and limitations

Analysis of the survey data and of reports made to regulatory agencies consists of event tabulations and summary calculations of percentages of occurrence for AE with a focus in the present summary on serious neurologic effects and deaths reported in canines. Statistical analysis was made for the severe AE of: death, seizures and convulsions, and ataxia, instability and imbalance by comparing the two‐tailed Z‐test differences between the Project Jake survey with those reported by the FDA and EMA. The number of reports represents the number of AE received for a particular drug and do not infer the relative toxicity between drugs. AE reports should not be used to calculate incidence rates or estimates of drug risk, since there is no accurate way to determine how many animals were actually given the drug, which is needed as the denominator in calculations of incidence and relative risk. Beyond the manufacturer's advertising and marketing materials, there is no indication of how many of the products have been sold. Furthermore, neither the FDA nor EMA address the total doses or treatments given in their data, and the package inserts do not delineate how many animals were treated and how many had serious AE (Tables 2, 3, 4, 7, 8; Appendix).

Since an AE may have been related to an underlying disease, use of other drugs at the same time, or other non‐drug related causes, these circumstances create uncertain causality between the adverse event and treatment with the drug. The frequency of reporting for a given product may vary over time and may be higher when the drug is newly marketed, or when media publicity occurs, thus underreporting generally occurs with most AE reporting systems.

Regulatory AE reporting systems as well as the Project Jake survey are all voluntarily reported with inherent biases weighted towards adverse effects, since few pet caregivers/owners or veterinarians have cause to report the absence of side effects. However, the Project Jake survey included questions and responses about drug treatment with or without the observation of AE, and thus it provided a population of responders registering treatment without AE. In fact, 36.1% of the Project Jake survey respondents indicated that they did not see any AEs and another 8.2% were unsure (Table 1). Also, because the FDA CVM and EMA ADE reporting systems depend upon the voluntary reporting of adverse clinical events by veterinarians and animal caregiver/owners, as well as the mandatory reporting of AE by manufacturers, aggregate AE reports potentially may include duplicative reported events.

**Table 1:** Summary of Project Jake Survey Respondent Numbers and Treatments

Summary: Isoxazoline Survey ‐ August 1–31, 2018		
Total Respondents:	2,751	
Respondents who gave their dog any flea treatment:	1594	57.9%
Respondents who gave their dog an isoxazoline treatment:	1,325	48.2%
Which Isoxazoline?		
Fluralaner (Bravecto)	911	68.8%
Afoxolaner (Nexgard)	342	25.8%
Sarolaner (Simparica)	72	5.4%
Did the dog experience a reaction to any treatment?		
Yes	1,062	66.6%
No	576	36.1%
Unsure	130	8.2%
Respondents whose dogs experienced a reaction to Isoxazolines:		
Fluralaner (Bravecto)	791	86.8%
Afoxolaner (Nexgard)	235	68.7%
Sarolaner (Simparica)	44	61.1%

Products	*N*	% of Responses
Bravecto (fluralaner)	911	33.12%
Nexgard (afoxolaner)	342	12.43%
Simparica (sarolaner)	72	2.62%
Advantage/Advantage II/ Advantage Multi	26	0.95%
Advantix/Advantix II	59	2.14%
Advocate	13	0.47%
Comfortis	29	1.05%
Frontline/Frontline Plus	198	7.20%
Kin and kind	19	0.69%
More than One Product	45	1.64%
Other (exclusively)	90	3.27%
Revolution	22	0.80%
Sentinel	23	0.84%
Seresto collar	36	1.31%
Trifexis	19	0.69%
Vectra/Vectra 3D	16	0.58%
Wondercide	15	0.55%

Note

Fluralaner, C22H17Cl2F6N3O3 ‐ PubChem;

Afoxolaner, C26H17ClF9N3O3 ‐ PubChem;

Sarolaner, C23H18Cl2F4N2O5S ‐Pub Chem

Lotilaner, C20H14Cl3F6N3O3S‐PubChem

## 3 RESULTS

**Figure 1:**
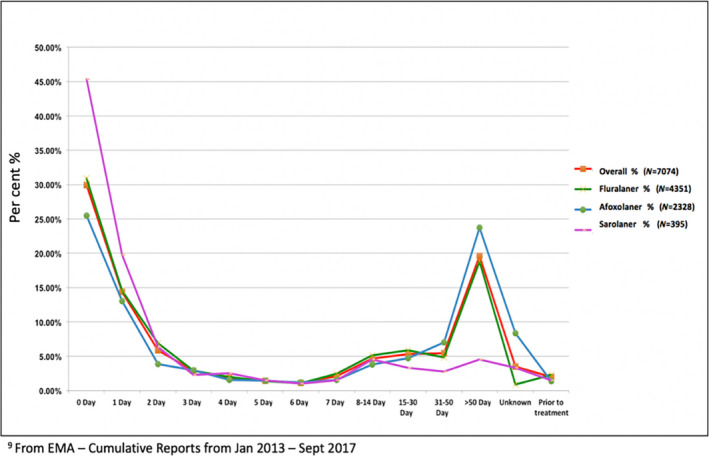
EMA time from dosing to adverse event

The canine products with the largest number of survey responses from the FDA, EMA and Project Jake survey were afoxolaner (Nexgard ®, Frontline Vet Labs™, Div. Merial Inc., Duluth, GA) and fluralaner (Bravecto®, Merck Animal Health, Madison, NJ) (Tables 1, 2, 3, 4, 5, 6, 7, 8) Other isoxazoline products that were reported in smaller numbers were sarolaner (Simparica™, Zoetis Inc, Kalamazoo, MI) and lotilaner (Credelio™, Elanco US, Greenfield, IN) (Tables 1, 2, 3, 4, 5, 6, 7, 8, 9, Figure 1). Another reported product in both the FDA and EMA AE reports was spinosad, a different class of drug (Comfortis ®, Elanco US, Greenfield, IN). (Tables 2 and 8).

**Table 2:** Summary of FDA^1‐8^ and EMA^9^ Adverse Event Reports for Side Effects of Isoxazolines

2018 Label Side Effects Listed^a^ Afoxolaner (Nexgard) Vomiting, decrease appetite, diarrhea, lethargy, polydipsia, flatulence; contains a caution about use in dogs with a history of seizures. Fluralaner (Bravecto) Vomiting, decrease appetite, diarrhea, lethargy, polydipsia, flatulence; label recently expanded to include seizures Sarolaner (Simparica) Vomiting, seizure, lethargy, diarrhea, tremor, ataxia; “may cause abnormal neurologic signs such as tremors, ataxia,... Seizures”

FDA Adverse Reaction Reports^1−8^ 32,374 Adverse Event Reports in Dogs with 801 Deaths and 1728 Seizures Product (Reports), Deaths/%, Seizures/% Afoxolaner (14,116), 341/2.4%, 981/6.9% Nexgard^1−8^ Fluralaner (16,896), 416/2.5%, 468/2.8% Bravecto^1−8^ Sarolaner (1,361), 44/3.2%, 279/20.5% Simparica^4−8^ Note: Lotilaner (Credelio) AE reports were not available via FOIA due to the newness of this drug Spinosad (Comfortis) AE reports not available at time of analysis

FOIA FDA: Total **Nexgard Reports** = 14,116 that contain 47,550 Events; Deaths = 341/14116 ( 2.4%); Seizures = 981/14116 ( 6.97%) Total **Bravecto Reports** = 16,896 that contain 45,924 Events; Deaths = 416/16896 (2.5%); Seizures = 468/16896 (2.8%) Total **Simparica Reports** = 1,361 that contain 5,977 Events; Deaths = 44/1361 (3.2%); Seizures = 279/1361 ( 20.5%)

^1–8^FOIA: CVM ADE Comprehensive Clinical Detail Report Listing, Cumulative Date Range:‐Jan‐2013 ‐ Sept‐2017.

^9^From EMA – cumulative reports from Jan 2013 ‐ Jan 2019.

^a^ Package Inserts are available in Appendix B.

**Table 3:** FDA Adverse Event Reports^1‐8^ for Side Effects of Isoxazolines

Event	Number and percent of sample population displaying reaction							
	Overall (*N* = 32,374)		Fluralaner (*N* = 16,896)		Afoxolaner (*N* = 14,116)		Sarolaner (*N* = 1,361)	
	*N*	%	*N*	%	*N*	%	*N*	%
Death	801	2.47%	416	2.50%	341	2.40%	44	3.20%
Seizure	1728	5.34%	468	2.80%	981	6.90%	279	20.50%
Shaking/Tremors/Ataxia	2,223	6.87%	600	3.60%	1,063	7.50%	560	41.10%
Behavioural Issues	9,266	28.62%	3,533	20.90%	5,098	36.10%	635	46.70%
Neurological/Cognitive	681	2.10%	275	1.60%	315	2.20%	91	6.70%
Muscular/Balance Issues	1778	5.49%	716	4.20%	879	6.20%	183	13.40%
Skin Problems/Itching	7,502	23.17%	1717	10.20%	5,563	39.40%	222	16.30%
Internal Bleeding	1,111	3.43%	509	3.00%	548	3.90%	54	4.00%
Anaemia	213	0.66%	127	0.80%	69	0.50%	17	1.20%
Vomiting/Nausea	13,251	40.93%	7,372	43.60%	5,347	37.90%	532	39.10%
Not Eating/Drinking	4,639	14.33%	1983	11.70%	2,366	16.80%	290	21.30%
Diarrhea	3,995	12.34%	1546	9.10%	2,195	15.50%	254	18.70%

**Table 4:** EMA Adverse Event Reports^9^ for Side Effects of Isoxazolines

EMA Event(s) Reported Jan 2013 – Sept 2017	Percent of sample population displaying reaction							
	Overall (*N* = 7,074)		Fluralaner (*N* = 4,351)		Afoxolaner (*N* = 2,328)		Sarolaner (*N* = 395)	
	*N*	%	*N*	%	*N*	%	*N*	%
Death	1,603	22.66%	1,025	23.56%	528	22.68%	50	12.66%
Seizure	2,140	30.25%	815	18.73%	1,087	46.69%	238	60.25%
Ataxia or Tremors	493	6.97%	271	6.23%	178	7.65%	44	11.14%
Behavioural Issues (i.e. Aggression, agitation, anxiety)	535	7.56%	297	6.83%	203	8.72%	35	8.86%
Loss of Motor Function, Limb Stiffness, Inability to Walk, etc.	531	7.51%	328	7.54%	160	6.87%	43	10.89%
Loss of Coordination/ Balance	98	1.39%	68	1.56%	25	1.07%	5	1.27%
Respiratory Issues	387	5.47%	265	6.09%	113	4.85%	9	2.28%
Vomiting	1857	26.25%	1,278	29.37%	515	22.12%	64	16.20%
Lethargy, lack of energy,	1,411	19.95%	954	21.93%	411	17.65%	46	11.65%
Loss of Appetite	1,371	19.38%	970	22.29%	364	15.64%	37	9.37%
Diarrhea	1,181	16.69%	864	19.86%	278	11.94%	39	9.87%

**Table 5:** Isoxazoline Serious Adverse Events in Project Jake Survey

Symptoms	Fluralaner (*N* = 791)		Afoxolaner (*N* = 235)		Sarolaner (*N* = 44)	
	*N*	%	*N*	%	*N*	%
Death	117	14.79%	28	11.91%	2	4.55%
Seizures/Convulsions	117	14.79%	28	11.91%	2	4.55%
Ataxia/Instability/Imbalance	116	14.66%	29	12.34%	6	13.64%
Shaking/Trembling	136	17.19%	36	15.32%	7	15.91%
Weakness	154	19.47%	37	15.74%	6	13.64%
Restlessness/Anxiety	144	18.20%	40	17.02%	5	11.36%
Abnormal Feces or Stools	120	15.17%	26	11.06%	3	6.82%
Lethargy/Depression	248	31.35%	57	24.26%	8	18.18%
Panting	144	18.20%	40	17.02%	7	15.91%
Decreased/No Appetite	224	28.32%	52	22.13%	7	15.91%
Itching/Scratching	119	15.04%	42	17.87%	2	4.55%
Vomiting	169	21.37%	49	20.85%	4	9.09%
Diarrhea	180	22.76%	47	20.00%	4	9.09%
Weight Loss	112	14.16%	21	8.94%	4	9.09%
Excessive Drinking	137	17.32%	24	10.21%	5	11.36%
Decreased Water Intake	66	8.34%	16	6.81%	3	6.82%
Excessive Urination	71	8.98%	14	5.96%	1	2.27%
Flatulence	31	3.92%	8	3.40%	0	0.00%
Hair Loss/Alopecia	48	6.07%	18	7.66%	2	4.55%
Other	47	5.94%	19	8.09%	4	9.09%
None	3	0.38%	0	0.00%	0	0.00%

**Table 6:** Correlation of Death With Neurological Symptoms in Project Jake Survey

Symptoms	Fluralaner (*N* = 791)		Afoxolaner (*N* = 235)		Sarolaner (*N* = 44)	
	*N*	%	*N*	%	*N*	%
Death	117	14.79%	28	11.91%	2	4.55%
Seizures/Convulsions	117	14.79%	28	11.91%	2	4.55%
Ataxia/Instability/Imbalance	116	14.66%	29	12.34%	6	13.64%
Shaking/Trembling	136	17.19%	36	15.32%	7	15.91%

Symptoms	*N*/% of dogs that died that also experienced the following NS					
	Fluralaner (*N* = 117)		Afoxolaner (*N* = 28)		Sarolaner (*N* = 2)	
	*N*	%	*N*	%	*N*	%
Seizures/Convulsions	36	30.77%	6	21.43%	2	100.00%
Ataxia/Instability/Imbalance	48	41.03%	13	46.43%	2	100.00%
Shaking/Trembling	41	35.04%	10	35.71%	1	50.00%

**Table 7:** Comparison of Project Jake Survey AE to Product Package Inserts^a^

Fluralaner Symptoms	Project Jake Survey		Package Insert	
	*N*	%	*N*	%
Decreased/No Appetite	224	28.32%	15	6.70%
Diarrhea	180	22.76%	11	4.90%
Excessive Drinking	137	17.32%	4	1.80%
Flatulence	31	3.92%	3	1.30%
Lethargy/Depression	248	31.35%	12	5.40%
Vomiting	169	21.37%	16	7.10%
Death	117	14.79%	Not reported	
Seizures/Convulsions	117	14.79%		
Shaking/Trembling	136	17.19%		
Ataxia/Instability/Imbalance	116	14.66%		
Weakness	154	19.47%		
Restlessness/Anxiety	144	18.20%		
Panting	144	18.20%		
Itching/Scratching	119	15.04%		
Abnormal Feces or Stools	120	15.17%		
Weight Loss	112	14.16%		
Decreased Water Intake	66	8.34%		
Excessive Urination	71	8.98%		
Hair Loss/Alopecia	48	6.07%		
**Total**	791	100%	224	100%

Afoxolaner Symptoms	Project Jake Survey		Package Insert	
	*N*	%	*N*	%
Decreased/No Appetite	52	22.13%	5	1.20%
Diarrhea	47	20.00%	13	3.10%
Lethargy/Depression	57	24.26%	7	1.70%
Vomiting	49	20.85%	17	4.10%
Dry/Flaky Skin	Not reported		13	3.10%
Death	29	12.34%	Not reported	
Seizures/Convulsions	28	11.91%		
Shaking/Trembling	36	15.32%		
Ataxia/Instability/Imbalance	26	11.06%		
Weakness	37	15.74%		
Restlessness/Anxiety	40	17.02%		
Panting	40	17.02%		
Itching/Scratching	42	17.87%		
Abnormal Faeces or Stools	26	11.06%		
Weight Loss	21	8.94%		
Excessive Drinking	24	10.21%		
Decreased Water Intake	16	6.81%		
Excessive Urination	14	5.96%		
Hair Loss/Alopecia	18	7.66%		
**Total**	235	100.00%	415	100.00%

^a^ Package Inserts are available in Appendix B.

**Table 8:** Summary of Serious Adverse Events Reported in FDA, EMA and Project Jake Surveys

FDA Serious Adverse Events Reports								
Events Reported	Number and Percent of Sample Population Displaying Reaction							
Jan 2013‐ Sept 2017	Overall (*N* = 32,374)		Fluralaner (*N* = 16,896)		Afoxolaner (*N* = 14,116)		Sarolaner (*N* = 1,361)	
Death	801	2.47%	416	2.50%	341	2.40%	44	3.20%
Seizure	1,728	5.34%	468	2.80%	981	6.90%	279	20.50%
Shaking/Tremors/Ataxia	2,223	6.87%	600	3.60%	1,063	7.50%	560	41.10%

Jake Survey Serious Adverse Events Results								
Events/Symptoms	Overall (*N* = 1,070)		Fluralaner (*N* = 791)		Afoxolaner (*N* = 235)		Sarolaner (*N* = 44)	
	*N*	%	*N*	%	*N*	%	*N*	%
Death	147	13.74%	117	14.79%	28	11.91%	2	4.55%
Seizures/Convulsions	147	13.74%	117	14.79%	28	11.91%	2	11.14%
Shaking/Tremors/Ataxia	493	43.2%	271	34.3%	178	75.7%	13	29.6%

EMA Serious Adverse Events Results								
Event(s) Reported Jan 2013–2019	Percent of Sample Population Displaying Reaction							
	Overall (*N* = 39,148)		Fluralaner (*N* = 10,172)		Afoxolaner (*N* = 20,902)		Sarolaner (*N* = 1917)	
Death	5,556	14.19%	2,408	23.7%	528	22.68%	250	13.04%
Seizure	6,272	16.02%	1,860	18.30%	1,087	46.69%	1,055	55.1%

					Credelio (*N* = 77)		Comfortis (*N* = 6,084)	
					*N*	%	*N*	%
Death^2^					22	28.60%	1882	30.9%
Seizure					21	27.60%	1,460	24.00%

**Table 9:** EMA^9^ Isoxazoline Treatment Relationship of Death & Seizure with Age

Dogs with Adverse Events (*N* = 7,074)			
Dogs that Died (*N* = 1603)		*N*	**%**
Age	<=1 Year	168	10.48%
	1–2.9 Years	111	6.92%
	3–4.9 Years	143	8.92%
	5–7.9 Years	286	17.84%
	>=8 Years	718	44.79%
	Unknown/Not Reported	177	11.04%

Dogs with Adverse Events (*N* = 7,074)			
Dogs that experienced Seizures (*N* = 2,140)		*N*	%
Age	<=1 Year	185	8.64%
	1–2.9 Years	266	12.43%
	3–4.9 Years	355	16.59%
	5–7.9 Years	457	21.36%
	>=8 Years	736	34.39%
	Unknown/Not Reported	141	6.59%

### 3.1 **FDA and EMA adverse reaction (AE) reports** (Tables 2‐4; Appendix)

The authors reviewed FDA and EMA reported AE available via public access of FOIA‐requested FDA AE reports (in Appendix; #1‐8) and the updated EMA AE reports (Appendix, #9). An analysis of these AE types is presented in Table 2 and the subsequent Tables (3–7), and includes analysis of FDA, EMA and Project Jake survey results with a particular focus on the most severe AE, namely, death and neurological effects (Tables 6‐9).

FDA reportable AE events from January 2013 to September 2017 are listed in Tables 2 and 3. These data show 32,374 reportable AE in dogs for this time period, with deaths (2.4 and 2.5%) and seizures (6.9 and 2.8%) for the isoxazolines afoxolaner (Nexgard ®, Frontline Vet Labs™, Div. Merial Inc., Duluth, GA) and fluralaner (Bravecto®, Merck Animal Health, Madison, NJ), respectively, while sarolaner (Simparica™, Zoetis Inc, Kalamazoo, MI) had 3.2% deaths and 20.5% seizures. Lotilaner (Credelio™, Elanco US, Greenfield, IN) and spinosad (Comfortis ®, Elanco US, Greenfield, IN) data were not available at the time of this analysis. Comparing the same time period (January 2013‐September 2017), EMA isoxazoline AE reports (7,074, Table 4) were approximately one‐fourth of the number of FDA reports, there were approximately 3 to 10 times the number of severe AE for these isoxazolines (Tables 3 and 4) shown in the FDA AE (32,374) reports. However, the EMA AE cumulative reportable events were updated in January 2019 and there are now 39,148 EMA AE reports from January 2013 to January 2019 (Table S1), which reflects about a fourfold increase from September 2017 to January 2019. During this extended reporting period, EMA data reflected notably higher serious AE for the four isoxazolines, ranging from 4.76% to 28.6% for deaths and 9.0 to 55.1% for seizure observations. Note that these EMA data included AE results for spinosad (6,080, Tables 8 and Table S1) with death (30.9%) and seizure (24%) rates markedly higher than serious AE rates for fluralaner, afoxolaner and sarolaner. The AE considered most serious with respect to neurological toxicity are listed at the top of Tables, 3–6, and the remaining reported AE are generally grouped by percentage occurrence and physiologic significance.

### 3.2 Serious AE findings from the FOIA‐requested CVM comprehensive clinical detail report listing (for comparable cumulative date range of January 2013 to September 2017)

These data are presented in Tables 3 and 8. Deaths for the fluralaner, afoxolaner and sarolaner isoxazolines in the FDA AE reports ranged from 2.5% to 3.2%. However, seizures, trembling and ataxia ranged from 2.8% to 7.5% for afoxolaner and fluralaner but were markedly higher for sarolaner at 20.50 and 41.1% (Tables 2, 3 and 8). Additionally, behavioural, cognitive and muscular AE observations for sarolaner were ~ 1.5 to 2 times higher than those noted for afoxolaner and fluralaner (Table 3).

Findings from the EMA AE reports for the same date range of January 2013 to September 2017 are presented in Table 4. There were 7,074 AE reported compared with the FDA reports of 32,374 in the same period; FDA reports showed about 3.4 to 6.1 times the number of EMA AE for these isoxazolines. The EMA AE considered most serious with regard to neurological toxicity are shown at the top of the Table 4, in similar placement to Table 3 for the FDA AE data. Of major significance was the fact that deaths in the EMA data were approximately 7 to 10 times higher than those reported in the corresponding time period to the FDA. Serious AE for seizure and tremor events in the EMA data were also notably higher than those reported to the FDA (Table 4). Furthermore, sarolaner reports were markedly higher than for afoxolaner and fluralaner, while the behavioural, cognitive and muscular AE observations in the EMA report data for sarolaner were similar to those noted for afoxolaner and fluralaner.

In comparing the updated cumulative data became available from the EMA for the extended period of January 2013 to January 2019. The overall number of EMA AE reports (39, 148) for afoxolaner, fluralaner and sarolaner in this update was essentially the same as those of the FDA events (32,374), as shown on Tables 2 and 3. Additionally, death and seizure reports for lotilaner and spinosad became available and are listed on Tables 2 and 8 for comparison. This larger EMA data set, which included an additional 15 months of reports, shows death and seizure events for afoxolaner comparable to the FDA findings with over 32,000 reported events. But, fluralaner and sarolaner EMA reported death and seizure events remained approximately 7 to 10 times higher than the reported FDA events. Remarkably, the death and seizure reports for lotilaner and spinosad were also about 10 times higher than those reported in the FDA data for the isoxazolines (Table 8).

A relationship between the occurrence of death and/or seizures was examined for the EMA cohort data from the January 2013 to September 2017 period versus January 2013 to January 2019 (Table S1). Comparing these two time periods, not only is there a substantial increase in AE reports, but the percentage of seizures and deaths is still remarkable even with a larger data set. The death rate range per isoxazoline type for 2013–2017 was 13%–24% versus 5%–31% for 2013–2019. The seizure rate range per isoxazoline type for 2013–2017 was 19%–60% versus 9%–55% for 2013–2019. The major difference in the two data sets is the type of isoxazoline used with the increase in afoxolaner use, and the introduction of new isoxazolines. These data indicated that not all dogs with seizures died, but the converse result could not be determined. With respect to pet age, serious AE showed death reported in about 60% of dogs older than 5 years with seizures in more than 50% of dogs older than 5 years (Table 9). An analysis of possible concurrent disease or health status was not possible for this data set. An analysis of the reported death and seizure AE associated with product dosage was inconclusive, although the data suggested that these serious AE occurred at most dosages and the Fluralaner dose of 1,000 mg appears to be an outlier, with 310 reports of seizures (30%) and 379 reports of death (38%), some of these could be the same animal, of which cannot be confirmed. (Table S2). The most serious AE as stated in the EMA cumulative reports were observed at 0–24 hr after the first dose, and then again after the second and third doses (Figure 1), which was consistent with the known isoxazoline pharmacokinetics of absorption and elimination following administration of oral chews (Letendre et al., 2014). Additionally, the pharmacokinetics and half‐life of isoxazolines were discussed by Drag et al. (2014).

### 3.3 Project Jake survey AE reports (Tables 1, 5, 6)

The Project Jake survey data results are shown in Tables 1, 5 and 6 and consolidated in Table 8, in comparison to the FDA and EMA data. Table 1 lists the survey findings for isoxazolines and an array of other topical flea and tick parasiticide products administered by survey responders; the number (N) of treated pets shows the percentages of the total number of responders (2,751). For the 12 topical formulations of flea and tick preventives listed in Table 1, there were 450 respondents that used them. Of these 450 responses, the number using them varied from 2.9% to 22.7%, with another 10% using a combination of them and 1.1% unsure what was used.

Tables 1 and 5 also show that of the 2,751 total Project Jake survey responders 57.9% (1594) had treated their pet dogs with a flea and tick product whereas 42.1% (1,157) of responders replied “No, Unsure, or failed to respond to this question”. Because of the nature of the survey question posed, we couldn't determine how many AE responders reported “Unsure” ‐ for the time of exposure to the AE. Responses in Table 1 that included dogs treated with an isoxazoline flea/tick product showed that 48.2% (1,325) were given either fluralaner (68.8%), afoxolaner (25.8%) or sarolaner (5.4%). Lotilaner data were not included in the responses for this survey since it was new to the market at that time. Of the 1594 dogs receiving any brand of flea & tick remedy 66.6% recorded some type of reported AE (Table 1). For dogs treated with an isoxazoline, 86.8% of fluralaner treated pets experienced a reaction; whereas 68.7% given afoxolaner and 61.1% of sarolaner treated dogs had a reaction following these treatments. Of those responders who treated their dogs with isoxazolines (1,325) 80.1% reported adverse effects in their pets. Serious AE were noted in 4.55 to 14.79% of animals given isoxazolines (Tables 5 and 6). Fluralaner and afoxolaner had reports of 11.91 to 14.79% seizures and deaths (Table 5). Sarolaner statistics were circumspect since the number of respondents was very limited.

The number of dogs with both seizures and death suggests a potential progression of symptoms as shown in Table 6. When examined for dogs that experienced seizures and death the data reveal that for fluralaner and afoxolaner approximately 20 to 46% of dogs that had seizures/tremors subsequently died. Necropsies were performed on two dogs with a pathological diagnosis of neurotoxicity determined in one and “not able to be determined” cause in the other one. These dogs also had tremors/shaking/ ataxia noted as early signs of neurotoxic effects. Approximately 54 to 80% of dogs that died did not have seizures/tremors/ataxia noted in the responses.

Serious AE responses for dogs receiving isoxazolines from the Project Jake survey are summarized in Table 5, and Table 6 lists the Project Jake survey AE for those dogs receiving isoxazolines that died after experiencing seizures or other neurological symptoms. However, not all dogs presenting with seizures, ataxia, instability, imbalance, shaking and trembling progressed to death (Table 6).

The number of AE reported by respondents in the Project Jake survey is shown in Table 7 compared to those AE listed in the manufacturer package inserts for fluralaner and afoxolaner. This tabulation demonstrates that no serious AE are listed in the inserts for either drug (Table 7). Serious neurological AE findings from the FDA, EMA and Project Jake survey are consolidated in Table 8. Although the number of AE reported to the FDA (32,374) and EMA (39,148) is essentially comparable, a 7 to 10 times higher occurrence of death and seizure was reported to the EMA from territories outside the United States. The source of AE reports should be compared, namely the percentage of reports received from veterinarians versus pet owners. The reports of death and seizure for lotilaner and spinosad are noteworthy with respect to product labels and inserts for potential neurological effects, as shown in the Appendix. The reported responses for the Project Jake survey for death and seizure observations were significantly higher than in the FDA dataset and comparable to the findings from the EMA reports (Deaths: EMA = 14.19%, Project Jake = 13.74%; Seizures: EMA = 16.02%, Project Jake = 13.74% (Tables 6 and 8).

### 3.4 Statistical analysis

The Project Jake survey percentages of AE were below those reported by the EMA but much higher than those reported by the FDA. However, the *p*‐values for the two‐tailed Z‐test differences between the Project Jake survey and fluralaner, afoxolaner and sarolaner for both the FDA and EMA reported AE were all < 0.00001. The statisticians in our group have indicated that an in‐depth statistical analysis of the rest of the survey findings would not be applicable to this type of survey.

## 4 DISCUSSION

The Project Jake survey results shown here differed from those of the FDA and EMA AE reports in several ways, namely: (1) Pet caregivers/owners surveyed included those that did not see any AE, as well as those that did report them, providing a more unbiased review of isoxazoline use; (2) Survey included observations pre‐ (3 months) and post‐drug use; (3) Questioned when AE occurred after use of the drug; (4) Questioned number of doses of drug(s) given before AE; (5) Other concurrent health issues and treatments given were requested; (6) Multiple AE were listed for each report; (7) Recovery information and/or follow‐ups were included.

The consolidated FDA, Project Jake and EMA findings (Table 8) showed notable differences between survey populations regarding the percentage of neurological toxicity and serious AE, and fatal effects. Statistical analysis of these serious AE showed highly significant differences between the findings of the Project Jake survey and those reported by the FDA and EMA. While the number of death and seizure AE reported by the EMA was 7 to 10 times higher than those reported to the FDA, the reported responses for the Project Jake survey for death and seizures fell in between those of the FDA and EMA but aligned more closely with the EMA results. Furthermore, the number of reported death and seizure AE for lotilaner and spinosad were considerably higher than suggested with respect to their product labelling for potential neurological effects (Table 2 and 8). Based on the results from the Project Jake survey and reviews of publicly available FDA and EMA AE reports demonstrating that cross‐species neurotoxicity exists, the following issues also require further attention: Recognition of human exposure risk as delineated in package inserts, and concerns that may arise from recent proposals to repurpose isoxazoline veterinary drugs for application to vector‐borne human diseases by treating humans (Miglianico et al., 2018). Furthermore, there is a very real potential for food‐chain associated AE, since fluralaner (as Exzolt) has recently been approved for treatment in poultry in Europe (Exzolt fluralaner for chickens: http://www.ema.europa.eu/ema/index.jsp?curl=pages/medicines/veterinary/medicines/004344/vet_med_000353.jsp&midnonbreakingspace=nonbreakingspaceWC0b01ac058008d7a8).

Our review of the FDA and EMA AE reports has generated a list of questions and safety concerns: A warning has been sent out in September 2018 by the US FDA to alert pet owners and veterinarians to be aware of the potential for neurological adverse events in dogs and cats when treated with drugs that are in the isoxazoline class. The FDA has since requested that manufacturers of isoxazoline products to include new label information to highlight neurological events because these events were seen consistently across the isoxazoline class of products. Subsequently the FDA has updated the alert with additional manufacturer information in April 2019, August 2019 and October 2019.

Based upon the FOIA FDA and EMA summary findings and implications, the death and seizure rate differences noted for the EMA were about 3–10 times higher than the FDA (Table 8). Were these FDA and EMA AE reports filed by veterinarians or by pet caregivers/owners or both? Additionally, analysis of the EMA data indicated that more than 60% of canine deaths and 55% of seizures were in dogs > 5 years of age (Table 9), but information regarding concomitant disease or other treatments was unavailable. Finally, no FDA or EMA data were available to determine whether most dogs that died also had preceding seizures, nor were we able to establish the time to death, and onset of or recovery from seizures. In that regard, the latest update from the FDA states “Isoxazoline products have been associated with neurologic adverse reactions, including muscle tremors, ataxia and seizures in some dogs and cats. Although most dogs and cats haven't had neurologic adverse reactions, seizures may occur in animals without a *prior history*.”

In the Project Jake survey, although not all dogs presenting with ataxia, instability, tremors and/or seizures progressed to death, for the dogs that died, about 21 to 31% also had seizures (Table 6). Survey responders did not indicate the recovery status from these neurological effects. However, several isolated reports (private communications with the authors of which three are veterinarians have indicated extended long‐term neurologic signs with incomplete amelioration).

The recent FDA Center for Veterinary Medicine (CVM) has a “Fact Sheet for *Pet Owners* and *Veterinarians* about Potential Adverse Events Associated with Isoxazoline Flea and Tick Products”. In this publication, the FDA is alerting pet owners and veterinarians of the potential for neurological adverse events in dogs and cats when treated with drugs that are in the isoxazoline class. However, the FDA considers products in the isoxazoline class to be safe and effective for dogs and cats. Although most dogs and cats have not experienced neurological AE reactions, the FDA states that seizures may occur in animals without a prior history (FDA,https://www.fda.gov/AnimalVeterinary/ResourcesforYou/AnimalHealthLiteracy/ucm620940.htm). The FDA is working with manufacturers of isoxazoline products to include new label information to highlight neurological events because these events have been seen consistently across the isoxazoline class of products (FDA, https://www.fda.gov/AnimalVeterinary/NewsEvents/CVMUpdates/ucm620934.htm). Changes in Package labelling since September 2018 are contained in the Appendix.

In conclusion, isoxazolines exhibit killing activity through effects on the insect nervous system. Prior reports (Gassel et al., 2014) showed that the isoxazoline fluralaner did not demonstrate any inhibitory action on the GABACl channels of rats and suggested that fluralaner is a potent arthropod ‐ specific GABACl channel inhibitor.

However, FDA and EMA AE reports and Project Jake survey evidence consistently demonstrate in three separate, distinct data sets that the neurotoxicity is not arthropod‐specific, and that post‐marketing serious AE are much higher than in the IND submission studies. Thus, as a class of drugs, these data indicate that isoxazolines can work as intrinsic neurotoxins across species. As pre‐approval IND studies were done in only a limited number of animals that did not show neurotoxic serious AE, it is not surprising that higher frequencies of such AE were noted once they were commercialized and given to much larger populations.

The data also suggested notable differences in reporting of AE for the US versus European territories. This may reflect cultural tendencies and/or procedural methodologies for reporting of AE. Nevertheless, the national and international post‐market safety survey data summarized here indicate an immediate need for continued cross‐species studies and a critical review of product labelling by regulatory agencies and manufacturers. Moreover, this class of drugs had more serious AE than those reported in the package inserts. We believe that the FDA should consider additional changes to their criteria for AE observation, and define what would be needed for future clearances of this class of drugs.

## CONFLICT OF INTEREST

The authors disclose no financial interest nor conflicts‐of‐interest in the present study.

## AUTHOR CONTRIBUTIONS

**W. Jean Dodds:** Conceptualization; Formal analysis; Investigation; Methodology; Validation; Writing‐original draft; Writing‐review & editing. **Valerie Palmieri:** Conceptualization; Funding acquisition; Project administration; Writing‐original draft; Writing‐review & editing. **Judy Morgan:** Writing‐original draft. **Elizabeth Carney:** Writing‐original draft. **Herbert A Fritsche:** Writing‐original draft. **Jaclyn Jeffrey:** Validation; Writing‐original draft. **Rowan Bullock:** Formal analysis; Methodology; Writing‐original draft; Writing‐review & editing. **Jon P Kimball:** Formal analysis; Investigation; Methodology; Validation; Writing‐original draft; Writing‐review & editing.

## Supporting information

Appendix

Tables S1‐S2

## Project Jake Survey Instrument, 2018 (copy attached)

FOIA Requests: Afoxolaner^1‐8^/Fluralaner^1‐8^/Sarolaner^4‐8^ [Isoxazoline Side Effects FDA Adverse Reaction Reports]

1. FDA‐CVM FOIA Response 2015–4864, Nov 2015; CVM ADE Comprehensive Clinical Detail Report Listing, Cumulative Date Range: 01‐Jan‐2013 ‐thru‐ 17‐Jun‐2015
2. FDA‐CVM FOIA Response 2016–96, Jan 2016; CVM ADE Comprehensive Clinical Detail Report Listing, Cumulative Date Range: 18‐Jun‐2015 to 06‐Jan‐2016
3. FDA‐CVM FOIA Response 2016–2546, May 2016; CVM ADE Comprehensive Clinical Detail Report Listing, Cumulative Date Range: 07‐Jan‐2016 to 31‐Mar‐2016
4. FDA‐CVM FOIA Response 2016–6026, July 2016; CVM ADE Comprehensive Clinical Detail Report Listing, Cumulative Date Range: 04‐Apr‐2016 to 15‐Jul‐2016
5. FDA‐CVM FOIA Response 2016–8391, Nov 2016; CVM ADE Comprehensive Clinical Detail Report Listing, Cumulative Date Range: 16‐Jul‐2016 to 15‐Oct‐2016
6. FDA‐CVM FOIA Response 2017–963, Feb 2017; CVM ADE Comprehensive Clinical Detail Report Listing, Cumulative Date Range: 16‐Oct‐2016 to 26‐Jan‐2017
7. FDA‐CVM FOIA Response 2017–4111, May 2017; CVM ADE Comprehensive Clinical Detail Report Listing, Cumulative Date Range: 28‐Jan‐2017 to 2‐May‐2017
8. FDA‐CVM FOIA Response 2017–8858, Oct 2017; CVM ADE Comprehensive Clinical Detail Report Listing, Cumulative Date Range: 03‐May‐2017 to 30‐Sept‐2017
9. EMA – Committee for Medicinal Products for Veterinary Use (CVMP) meeting of 5–7 September 2017; Cumulative Reports from Jan 2013 – Sept 2017. https://www.ema.europa.eu/en/news/committee‐medicinal‐products‐veterinary‐use‐cvmp‐meeting‐5‐7‐september‐2017

## Excerpts from US package inserts state ‘SEIZURE’ as addition to isoxazoline labels after september 2018

Precautions

- **Afoxolaner** (Nexgard ®, Frontline Vet Labs™, Div. Merial Inc., Duluth, GA) is a member of the isoxazoline class. This class has been associated with neurological adverse reactions including tremors, ataxia and seizures. Seizures have been reported in dogs receiving isoxazoline class drugs, even in dogs without a history of seizures. Use with caution in dogs with a history of seizures or neurological disorders.
- **Fluralaner** (Bravecto®, Merck Animal Health, Madison, NJ). Important Safety Information (for Veterinarians): Use caution in dogs with a history of seizures. Seizures have been reported in dogs receiving fluralaner, even in dogs without a history of seizures. [in 2019 Canada label: Neurological disorders: convulsions, ataxia, muscle tremor]
- **Sarolaner** (Simparica™, Zoetis Inc, Kalamazoo, MI) may cause abnormal neurological signs such as tremors, decreased conscious proprioception, ataxia, decreased or absent menace, and/or seizures.
- **Lotilaner** (Credelio™, Elanco US, Greenfield, IN) The safe use of lotilaner in breeding, pregnant or lactating dogs has not been evaluated. Use with caution in dogs with a history of seizures.
- **Spinosad** (Comfortis ®, Elanco US, Greenfield, IN) Post Approval Experience (June 2009): The following adverse reactions are based on post‐approval adverse drug event reporting. The adverse reactions are listed in decreasing order of frequency: *vomiting, depression/lethargy, anorexia, ataxia, diarrhea, pruritus, trembling, hypersalivation and seizures*.
- A spinosyns class of insecticides, which are non‐antibacterial tetracyclic macrolides

All products have warnings against human exposure

## Canadian Fluralaner Package Insert (February 2019)

Prior to the FDA alert informing pet owners and veterinarians of potential for neurological adverse events for isoxazolines on September 21, 2018 product labelling did not contain precautionary or warning labelling of these AE. The excerpted statements are representative of (slightly) modified package labels containing cursory comments related to seizures. The Canadian fluralaner label version of 2019 lists seizure with the qualifier of occurrence rates of less than 1 in 10,000.

Fluralaner 2019 package labelling in Canada states: “The following adverse events have been reported rarely: 1(reported in at least 1 but not more than 10 animals in 10,000 animals exposed) and very rarely: 2(reported in less than 1 in 10,000 animals exposed) and are listed by body system, in decreasing order of frequency: Digestive tract disorders: vomiting, diarrhoea, hypersalivation, haemorrhagic diarrhoea Systemic disorders: lack of efficacy, lethargy, anorexia Skin and appendage disorders: pruritus, alopecia Neurological disorders: convulsions, ataxia, muscle tremor.” 
