# CORRIGENDUM

**DOI:** 10.1002/vms3.571

**Published:** 2021-07-16

In the article by Palmieri et al., the conflict of interest statement should read as below.

## CONFLICT OF INTEREST

A Class Action lawsuit related to the use and safety of isoxazoline parasiticides was filed on December 27, 2019 in New Jersey, while this manuscript was undergoing peer review. One of the article's co‐authors, Valerie Palmieri, is the Plaintiff. [PALMIERI, et al. v. INTERVET INC, Case No. .2:19‐cv‐22024‐JMV‐AME (D. N. J.).]”

## References

[vms3571-bib-0001] Palmieri, V., Dodds, W. J., Morgan, J., Carney, E., Fritsche, H. A., Jeffrey, J., Bullock, R., & Kimball, J. P. (2020). Survey of canine use and safety of isooxazoline parasiticides. Veterinary Medicine Science, 6(4), 933–945. 10.1002/vms3.285 32485788 PMC7738705

