# Supplementary material for: Survey of canine use and safety of isoxazoline parasiticides
Source: Vet Med Sci. 2020 Jun 2;6(4):933–45. doi: 10.1002/vms3.285 (PMC7738705; doi:10.1002/vms3.285)
Supplement: Supplementary file 1 — Appendix [file VMS3-6-933-s001.docx]

**APPENDIX D – Supplemental Tables**

**TABLE 10 –** EMA^9^ adverse event reports for side effects of isoxazolines for

time periods Jan 2013 –Sept 2017 and Jan 2013 – Jan 2019

| **EMA Event(s) Reported**  **Jan 2013 – Sept 2017** | **Percent of Sample Population Displaying Reaction** | | | | | | | |
| --- | --- | --- | --- | --- | --- | --- | --- | --- |
|  | **Overall (N=7074)** | | **Fluralaner (N=4351)** | | **Afoxolaner (N=2328)** | | **Sarolaner (N=395)** | |
|  | **N** | **%** | **N** | **%** | **N** | **%** | **N** | **%** |
| **Death^1^** | 1603 | 22.66% | 1025 | 23.56% | 528 | 22.68% | 50 | 12.66% |
| **Seizure** | 2140 | 30.25% | 815 | 18.73% | 1087 | 46.69% | 238 | 60.25% |

| **Event(s) Reported**  **Jan 2013 – Jan 2019** | **Percent of Sample Population Displaying Reaction** | | | | | | | |
| --- | --- | --- | --- | --- | --- | --- | --- | --- |
|  | **Overall (N=32992)** | | **Fluralaner (N=10172)** | | **Afoxolaner (N=20903)** | | **Sarolaner (N=1917)** | |
|  | **N** | **%** | **N** | **%** | **N** | **%** | **N** | **%** |
| **Death^2^** | 3652 | 11.07% | 2408 | 23.67% | 994 | 4.76% | 250 | 13.04% |
| **Seizure** | 6272 | 16.0% | 1860 | 18.3% | 1876 | 9.0% | 1055 | 55.1% |
|  | | | | |  | | | |
|  |  |  |  |  | **Credelio (N=77)** | | **Comfortis (N=6084)** | |
|  |  |  |  |  | **N** | **%** | **N** | **%** |
| **Death^2^** |  | | | | 22 | 28.6% | 1882 | 30.9% |
| **Seizure** |  | | | | 21 | 27.6% | 1460 | 24.0% |

| **Dogs that experienced Seizures (N=2140)** | | | |
| --- | --- | --- | --- |
|  | **Dosage** | **N** | **%** |
| **Fluralaner/**  **Bravecto** | **1400 mg** | 62 | **7.61%** |
|  | **1000 mg** | 310 | **38.04%** |
|  | **750 mg** | 0 | **0.00%** |
|  | **500 mg** | 145 | **17.79%** |
|  | **250 mg** | 200 | **24.54%** |
|  | **112.5 mg** | 84 | **10.31%** |
|  | **Unknown** | 14 | **1.72%** |
| **Afoxolaner/**  **NexGard** | **136 mg** | 288 | **26.49%** |
|  | **68 mg** | 304 | **27.97%** |
|  | **28.3 mg** | 303 | **27.87%** |
|  | **11.3 mg** | 158 | **14.54%** |
|  | **Unknown** | 34 | **3.13%** |
| **Sarolaner/**  **Simparica** | **320 mg** | 1 | **0.42%** |
|  | **240 mg** | 1 | **0.42%** |
|  | **160 mg** | 1 | **0.42%** |
|  | **130 mg** | 1 | **0.42%** |
|  | **120 mg** | 12 | **5.04%** |
|  | **90 mg** | 1 | **0.42%** |
|  | **80 mg** | 67 | **28.15%** |
|  | **40 mg** | 41 | **17.23%** |
|  | **20 mg** | 47 | **19.75%** |
|  | **15 mg** | 1 | **0.42%** |
|  | **10 mg** | 17 | **7.14%** |
|  | **5 mg** | 9 | **3.78%** |
|  | **Unknown** | 39 | **16.39%** |

**TABLE 11** – EMA^9^ isoxazoline relationship of side effects and dosage

| **Dogs that Died (N=1603)** | | | |
| --- | --- | --- | --- |
|  | **Dosage** | **N** | **%** |
| **Fluralaner/**  **Bravecto** | **1400 mg** | 93 | **9.07%** |
|  | **1000 mg** | 379 | **36.98%** |
|  | **750 mg** | 0 | **0.00%** |
|  | **500 mg** | 207 | **20.20%** |
|  | **250 mg** | 221 | **21.56%** |
|  | **112.5 mg** | 97 | **9.46%** |
|  | **Unknown** | 28 | **2.73%** |
| **Afoxolaner/**  **NexGard** | **136 mg** | 125 | **23.67%** |
|  | **68 mg** | 124 | **23.48%** |
|  | **28.3 mg** | 144 | **27.27%** |
|  | **11.3 mg** | 85 | **16.10%** |
|  | **Unknown** | 50 | **9.47%** |
| **Sarolaner/**  **Simparica** | **320 mg** | 0 | **0.00%** |
|  | **240 mg** | 0 | **0.00%** |
|  | **160 mg** | 0 | **0.00%** |
|  | **130 mg** | 0 | **0.00%** |
|  | **120 mg** | 5 | **10.00%** |
|  | **90 mg** | 0 | **0.00%** |
|  | **80 mg** | 11 | **22.00%** |
|  | **40 mg** | 4 | **8.00%** |
|  | **20 mg** | 8 | **16.00%** |
|  | **15 mg** | 0 | **0.00%** |
|  | **10 mg** | 4 | **8.00%** |
|  | **5 mg** | 1 | **2.00%** |
|  | **Unknown** | 17 | **34.00%** |
